# Case Report: Efgartigimod demonstrates significant clinical efficacy in double seropositive myasthenia gravis: a case report of a rare variant and analysis of pathomechanisms

**DOI:** 10.3389/fimmu.2025.1610738

**Published:** 2025-10-13

**Authors:** Xiaohui Huang, Yue Wan, Yu Chen, Keqi Lei

**Affiliations:** ^1^ Jianghan University, Wuhan, China; ^2^ Hubei Provincial Third People’s Hospital (Zhongshan Hospital), Wuhan, Hubei, China

**Keywords:** efgartigimod, double seropositive, myasthenia gravis, Fc receptor inhibitors, case report

## Abstract

Double Seropositive Myasthenia Gravis (DSP-MG), a rare variant of Myasthenia Gravis (MG), is defined by the simultaneous presence of both anti-acetylcholine receptor (AChR) antibodies and anti-muscle-specific tyrosine kinase (MuSK) antibodies in the serum of affected individuals. Currently, no standardized therapeutic protocol exists for DSP-MG due to its scarcity and clinical heterogeneity. Herein, we report a case of a 68-year-old female patient with DSP-MG who showed significant clinical improvement during an acute exacerbation after treatment with the FcRn antagonist efgartigimod, following the failure of conventional therapy. After a cycle of efgartigimod treatment, complete resolution of myasthenic symptoms was observed. During the 6-month follow-up, with sustained clinical remission and attainment of Minimal Manifestation Status (MMS). This case represents the first documented use of efgartigimod in a DSP-MG patient, providing preliminary clinical evidence for its potential efficacy in this rare and poorly understood subtype. Our findings contribute to the limited literature on DSP-MG and suggest that FcRn inhibition may offer a viable treatment option where conventional therapies fail. Efgartigimod may represent a potential therapeutic agent for DSP-MG.

## Introduction

Double Seropositive Myasthenia Gravis (DSP-MG) is a rare subtype of Myasthenia Gravis (MG) characterized by the simultaneous presence of anti-acetylcholine receptor (AChR) antibodies and muscle-specific tyrosine kinase (MuSK) antibodies (1). Its clinical features include involvement of a wide range of muscle groups, aggressive disease progression, and a high predisposition to myasthenic crises. Compared with AChR antibody-positive MG, its clinical features include involvement of a wide range of muscle groups, aggressive disease progression, and a high predisposition to myasthenic crises (2). Most cases occur sporadically, with only 28 documented cases worldwide as of 2025.

Currently, there is no universally recognized effective treatment regimen for DSP-MG. primarily due to significant differences in the pathogenic mechanisms mediated by distinct antibodies. AChR antibodies predominantly damage the postsynaptic membrane through complement-dependent cytotoxicity, while MuSK antibodies disrupt synaptic structural stability via non-complement-dependent mechanism. This mechanistic heterogeneity poses substantial clinical challenges in managing DSP-MG. Such divergent pathogenesis necessitates tailored therapeutic approaches. Cholinesterase inhibitors can improve the symptoms of MG patients with positive AChR antibodies, but they may be ineffective or even exacerbate the condition positive MuSK antibodies (3). Conversely, complement-targeted therapies (such as eculizumab) can alleviate the pathogenic effects of AChR antibodies, yet their therapeutic impact remains minimal in MuSK-MG (4). In addition, monoclonal antibodies (such as rituximab) have demonstrated more pronounced efficacy in MuSK-MG. In contrast, patients with AChR-MG exhibit a less consistent and weaker response, indicating that the therapeutic effects of rituximab in this subgroup require further investigation (5). Therefore, finding effective drugs for treating DSP-MG is an urgent task at present.

Efgartigimod is a targeted FcRn antagonist that promotes the clearance of pathogenic antibodies by blocking the interaction between IgG and FcRn (6).The drug has been approved for the treatment of MG patients with positive AChR antibodies, but its efficacy in other subtypes of MG, such as MG patients with positive MuSK antibodies and DSP-MG, remains unknown (7). However, emerging case reports have documented efgartigimod’s off-label use in diverse MG presentations, including MG patients with positive MuSK antibodies, refractory MG, and ocular myasthenia gravis (8–11). This case represents the first application of efgartigimod in a DSP-MG patient, demonstrating the effectiveness of efgartigimod in treating DSP-MG and providing preliminary evidence.

## Case description

**Figure 1:**
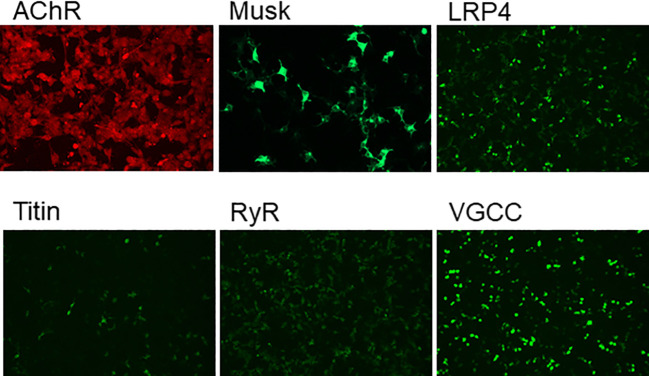
The presence of myasthenia gravis-related antibodies in the serum was confirmed by cell-based flow cytometric immunofluorescence assay, revealing a MuSK antibody titer of 1:320 and an AChR antibody titer of 1:32.

A 68-year-old female was admitted to our hospital in October 2024, due to progressive worsening of diplopia, dysarthria accompanied by dysphagia over 12 months. The initial clinical manifestations were mild dizziness and general fatigue. Subsequently, the patient developed progressive ptosis accompanied by diplopia and an ataxic gait. Four months later, bulbar palsy emerged, manifested as dysarthria and dysphagia. Notably, the symptoms fluctuated, typically worsening in the evening and improving in the morning. The patient was previously healthy with no personal or family history of genetic, autoimmune, or neurological disorders. Neurological examination revealed dysarthria, disappearance of bilateral pharyngeal reflexes, bilateral ptosis, limited abduction, impaired ocular motility (limited abduction and adduction) by downbeat nystagmus, air leakage during cheek puffing, weakened bilateral tendon reflexes, and a score of 3 in the Kubota Water Drinking Test. The results of laboratory examinations (complete blood cell count, urinalysis, liver and kidney function tests, electrolyte tests, coagulation function tests, homocysteine level test, lipid profile, blood glucose level test, thyroid function tests, antinuclear antibody spectrum, antineutrophil cytoplasmic antibody spectrum, routine and biochemical examination of cerebrospinal fluid, and other autoantibody tests) were normal. The edrophonium test was positive, supporting a diagnosis of MG. Serum anti-MuSK antibody (+) 1:320 and serum anti-AChR antibody (+) 1:32 (**Figure 1**) [detected using the cell-based flow cytometric immunofluorescence assay (CBA) method]. Electromyography (EMG) showed a decrement in the amplitude of low-frequency stimulation of the left accessory nerve. CT and MRI of the brain and cervical spine showed no significant structural abnormalities. Chest CT scan ruled out thymoma, and laryngoscopy excluded organic lesions in the pharynx and larynx. Based on these findings, myasthenia gravis was diagnosed (Myasthenia Gravis Foundation of America IIb, MGFA IIb), with a Quantitative Myasthenia Gravis (QMG) score of 9(double vision: spontaneous, score 3; ptosis: spontaneous, score 3; dysphagia: moderate,score 2,; dysarthria:mild,score 1) and MG-ADL score was 13.

**Figure 2:**
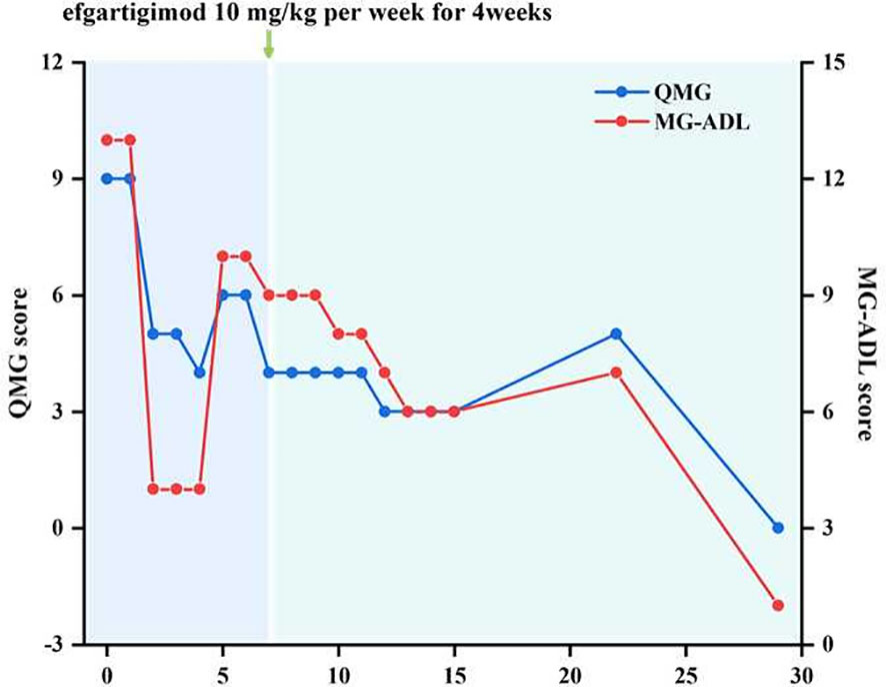
Trends in QMG and MG-ADL scores over the disease course.

At first, we administered pyridostigmine bromide 60 mg orally three times a day, steroids 20 mg orally once a day, and tacrolimus orally 4.5 mg a day. After one week of treatment, the patient’s symptoms showed some improvement while developing side effects such as intestinal colic and sialorrhea. On October 25, 2024, the patient developed new-onset nocturnal dyspnea and palpitations. Therefore, starting from October 27, 2024, the patient was given efgartigimod at a dose of 10 mg/kg intravenously once a week for 4 consecutive weeks. Two weeks after receiving Efgartigimod treatment, the patient’s dysphagia and dysarthria significantly improved compared with the previous condition. Four weeks later, the patient’s myasthenia-related symptoms completely improved. The Quantitative Myasthenia Gravis Score (QMG Score) decreased to 0 points, and the Myasthenia Gravis Activities of Daily Living Score (MG-ADL Score) decreased to 1 point (**Figure 2**). During the current 6-month follow-up of the patient, the trough concentration of tacrolimus in the serum was maintained at 6.2 ng/mL, and the clinical symptoms completely disappeared, achieving the Minimal Manifestation Status (MMS).

## Discussion

**Figure 3:**
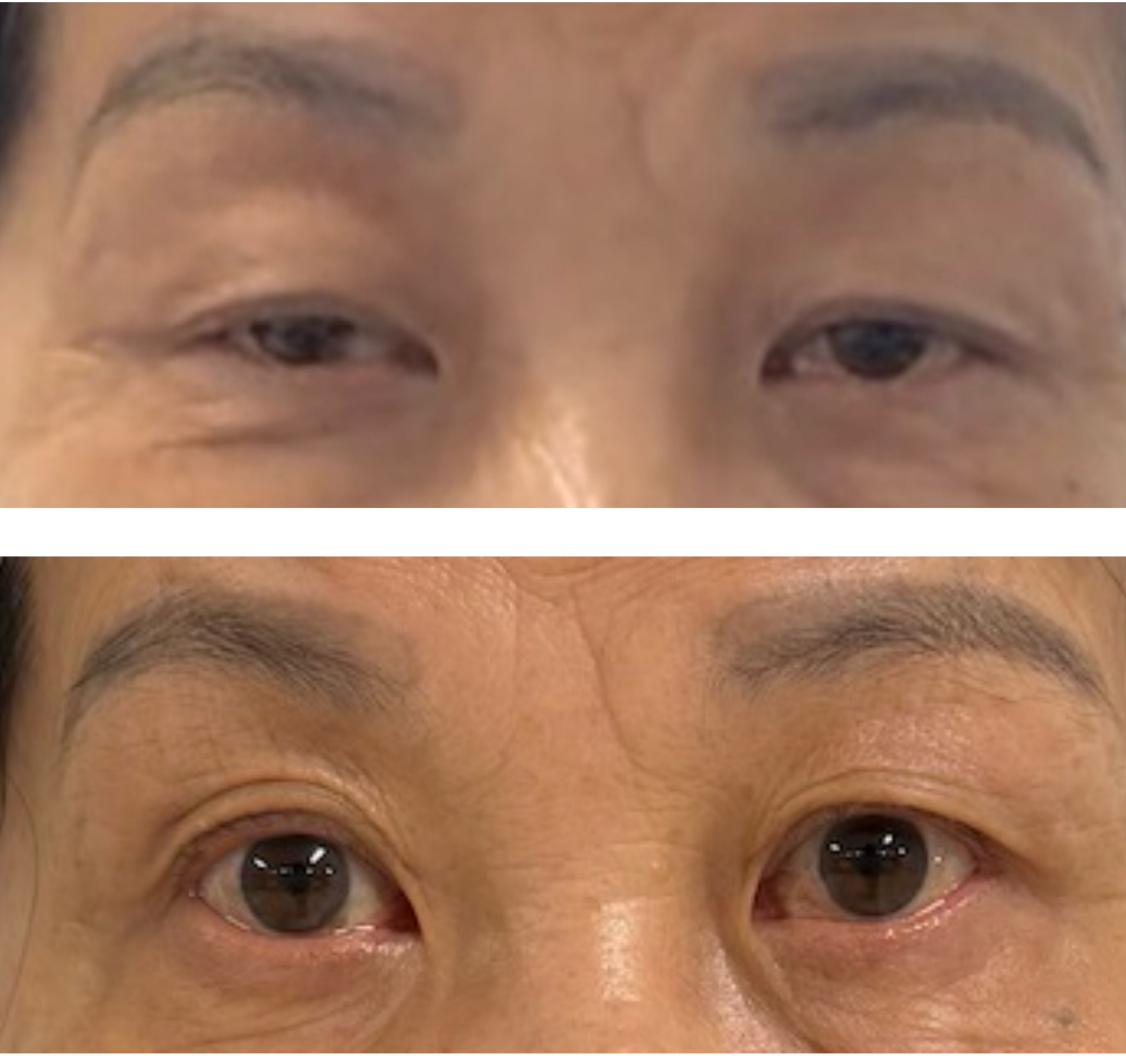
The top panel in the figure shows the images of the patient's bilateral eyelids before Efgartigimod administration, and the bottom panel presents the eyelid images after the completion of one treatment cycle, demonstrating improvement in ptosis (eyelid drooping) symptoms.

We report a clinically instructive case of double-seropositive myasthenia gravis (DSP-MG) in an elderly female patient who exhibited an excellent therapeutic response to efgartigimod (**Figure 3**). The disease course began with mild dizziness and progressed insidiously over twelve months to develop hallmark MG manifestations including diplopia and dysphagia. This subacute-onset pattern predominantly involved cranial nerve-innervated muscle groups, lacking classical myasthenic features such as skeletal muscle fatigability or ptosis/limb weakness that typically manifest in broader disease phenotypes. This cranial nerve-predominant pattern, combined with seropositivity for both AChR and MuSK antibodies, suggests a distinct clinical variant of double-seropositive MG. The clinical presentation mimicked neurological disorders including cerebrovascular events, central nervous system infections, and mitochondrial cytopathy, contributing to diagnostic delay.

The current mechanism of DSP-MG has not been fully elucidated. Current evidence suggests that its pathogenesis is likely mediated by cross-immunoreactivity induced by antigenic epitope spreading at the neuromuscular junction. In generalized myasthenia gravis with positive AChR autoantibodies, IgG1 and IgG3 autoantibodies bind to AChR, disrupt the transmission between neurons and muscles by activating the classical complement pathway, and block the binding of acetylcholine to AChR (12). In myasthenia gravis mediated by positive MuSK autoantibodies, IgG4 autoantibodies bind to the MuSK receptor and functionally block the transmission of neuromuscular signals (13). IgG4 molecules can exchange Fab segments to reduce the formation of immune complexes, thus evading the inhibition of the inflammatory response of immune complexes by traditional immunosuppressants (14). The dual-antibody effect in double seropositive myasthenia gravis (DSP-MG) likely represents a synergistic pathogenic mechanism rather than a simple additive effect. Emerging evidence suggests that AChR and MuSK antibodies may form a positive feedback loop via epitope spreading: AChR antibodies damage the structure of the postsynaptic membrane through complement activation, exposing the hidden MuSK epitopes; while MuSK antibodies further prevent the aggregation signal cascade of AChR by interfering with the agrin-LRP4-MuSK signaling pathway (15). This epitope spreading effect may be an important mechanism for the poor response of DSP-MG to conventional immunotherapy (16). Therefore, the clinical manifestations of DSP-MG are highly heterogeneous, causing significant challenges for clinical treatment.

In this case, we initially administered oral pyridostigmine bromide and steroids to the patient, but there was no obvious improvement in the patient’s condition. Instead, side effects such as intestinal colic and sialorrhea occurred. The limited therapeutic efficacy of cholinesterase inhibitors in MuSK-associated myasthenia gravis may be attributed to their mechanism of action. While these agents effectively inhibit acetylcholinesterase activity at the neuromuscular junction, they demonstrate significantly reduced clinical benefit in MuSK-positive MG, as the primary pathophysiology involves antibody-mediated disruption of MuSK-dependent postsynaptic signaling rather than solely acetylcholine deficiency. The patient subsequently developed nocturnal dyspnea, raising concern for impending myasthenic crisis. After systematically excluding cardiovascular causes and considering the risk of myasthenia gravis crisis, we immediately initiated the treatment with efgartigimod and tacrolimus. This was because, prior to this, efgartigimod had not only shown therapeutic value in MG patients with positive AChR antibodies but also exhibited significant response characteristics in a series of special subgroups of MG, such as MG patients with positive MuSK antibodies, isolated ocular myasthenia gravis, and triple-seronegative MG (8–10, 17). Moreover, according to the latest German guidelines for myasthenia gravis, neonatal Fc receptor (FcRn) antagonists are a an optimal therapeutic choice for patients experiencing acute MG exacerbations requiring rapid antibody depletion (18). While both intravenous immunoglobulin (IVIg) and plasma exchange (PLEX) demonstrate rapid therapeutic efficacy in myasthenic crisis, PLEX carries significant procedural limitations. The requirement for central venous access introduces inherent risks, including catheter-related infections (reported in 5-15% of cases) and thrombosis (occurring in 3-10% of procedures) (19). Moreover, the invasive nature of vascular access and need for specialized equipment often limit its use to tertiary care centers. In contrast, IVIg administration avoids these vascular complications but presents other challenges including volume overload and rare but serious adverse effects such as aseptic meningitis or hemolytic reactions. In comparison, although intravenous immunoglobulin (IVIg) and plasma exchange (PLEX) have also been proven to provide a rapid therapeutic response, plasma exchange requires central venous catheterization, and the vascular access is invasive. There are also non-negligible problems related to the venous access, such as related infections and thrombosis (19). As a blood product, IVIg always faces the problems of a lack of blood donors and potential infection risks. Targeted complement inhibitors represented by rituximab are a relatively effective treatment option for DSP-MG, but their immunological effects gradually appear after the depletion of circulating B cells and cannot achieve a rapid onset of action. In addition, studies have found that compared with other complement inhibitors (such as eculizumab), efgartigimod can provide a more flexible treatment cycle, fewer side effects, and better efficacy in a wider range of MG subtypes (such as ACHR-) (20).Considering that the patient was an elderly female, in order to balance treatment safety and minimize adverse reactions, we we prioritized both safety and efficacy in selecting efgartigimod to treat the acute exacerbation of DSP-MG. After treatment, the patient also showed amazing clinical efficacy.

This case represents the first reported application of efgartigimod in a DSP-MG patient, demonstrating its therapeutic efficacy and providing preliminary clinical evidence. The utilization of efgartigimod in DSP-MG management not only significantly reduced risks associated with invasive interventions but also achieved rapid reduction of pathogenic antibody levels. This study has several important limitations that warrant consideration. First, the extreme rarity of DSP-MG restricted our analysis to a single case, which precludes broad conclusions about treatment efficacy. Second, the observed therapeutic success may reflect unique patient characteristics (e.g., specific antibody titers, immunological profile, or comorbidities) rather than universal treatment effects. Third, without a controlled comparison group, we cannot exclude the possibility of spontaneous improvement. These constraints significantly limit the generalizability of our findings and highlight the need for larger, multicenter studies to validate efgartigimod’s efficacy in DSP-MG populations.

While the rarity of DSP-MG inherently limits our ability to validate these findings through large-scale studies, this case retains important clinical implications. It provides preliminary evidence that FcRn antagonists may represent an optimal salvage therapy for refractory MG cases involving multiple pathogenic antibodies, particularly in clinical settings demanding: (1) rapid immunomodulation, (2) avoidance of invasive procedures, and (3) minimization of treatment-related comorbidities. These observations warrant further investigation through international registry studies or collaborative case series to better define the role of FcRn inhibition in complex MG subtypes.

## Data Availability

The original contributions presented in the study are included in the article/**Supplementary Material**. Further inquiries can be directed to the corresponding author.

## References

[1] Zouvelou V Michail M Belimezi M Zisimopoulou P. Subunit specificity of the acetylcholine receptor antibodies in double seropositive myasthenia gravis. Muscle Nerve. (2021) 63:E36-E37. doi: 10.1002/mus.27177, PMID: 33471417

[2] Zhang J Chen Y Chen J Huang X Wang H Li Y. AChRAb and MuSKAb double-seropositive myasthenia gravis: a distinct subtype? Neurol Sci. (2021) 42:863–9. doi: 10.1007/s10072-021-05042-3, PMID: 33438140 PMC7870615

[3] Ricciardi R Latini E Guida M Koneczny I Lucchi M Maestri M. Acetylcholinesterase inhibitors are ineffective in MuSK-antibody positive myasthenia gravis: Results of a study on 202 patients. J Neurol Sci. (2024) 461:123047. doi: 10.1016/j.jns.2024.123047, PMID: 38759248

[4] Mantegazza R Antozzi C. From traditional to targeted immunotherapy in myasthenia gravis: prospects for research. Front Neurol. (2020) 11:981. doi: 10.3389/fneur.2020.00981, PMID: 32982957 PMC7492201

[5] Litchman T Roy B Kumar A Sharma A Njike V Nowak RJ. Differential response to rituximab in anti-AChR and anti-MuSK positive myasthenia gravis patients: a single-center retrospective study. J Neurol Sci. (2020) 411:116690. doi: 10.1016/j.jns.2020.116690, PMID: 32028072

[6] Menon D Bril V. Pharmacotherapy of generalized myasthenia gravis with special emphasis on newer biologicals. Drugs. (2022) 82:865–87. doi: 10.1007/s40265-022-01726-y, PMID: 35639288 PMC9152838

[7] Howard JF Bril V Vu T Karam C Peric S Margania T. Safety, efficacy, and tolerability of efgartigimod in patients with generalised myasthenia gravis (ADAPT): a multicentre, randomised, placebo-controlled, phase 3 trial. Lancet Neurol. (2021) 20:526–36. doi: 10.1016/S1474-4422(21)00159-9, PMID: 34146511

[8] Ma T Zhu Y Zhu R. Case report: efgartigimod is a novel therapeutic option for ocular myasthenia gravis: a report of 2 cases. Front Immunol. (2024) 15:1497398. doi: 10.3389/fimmu.2024.1497398, PMID: 39872535 PMC11769809

[9] Zhu G Ma Y Zhou H Nie X Qi W Hao L. Case report: Rapid clinical improvement in acute exacerbation of MuSK-MG with efgartigimod. Front Immunol. (2024) 15:1401972. doi: 10.3389/fimmu.2024.1401972, PMID: 38911858 PMC11190065

[10] Shi F Chen J Feng L Lai R Zhou H Sun X. Efgartigimod treatment in patients with anti-MuSK-positive myasthenia gravis in exacerbation. Front Neurol. (2024) 15:1486659. doi: 10.3389/fneur.2024.1486659, PMID: 39628891 PMC11611843

[11] Tanaka K Yoshikawa M Inoue Y Tsumura K Hoshino Y Shichijo C. Efgartigimod successfully ameliorated acute exacerbation of myasthenia gravis with anti-muscle-specific kinase antibodies. Intern Med. (2024) 64:3726–24. doi: 10.2169/internalmedicine.3726-24, PMID: 39111891 PMC11986312

[12] Howard JF. Myasthenia gravis: the role of complement at the neuromuscular junction. Ann N Y Acad Sci. (2018) 1412:113–28. doi: 10.1111/nyas.13522, PMID: 29266249

[13] Burden SJ Yumoto N Zhang W. The role of MuSK in synapse formation and neuromuscular disease. Cold Spring Harb Perspect Biol. (2013) 5:a009167. doi: 10.1101/cshperspect.a009167, PMID: 23637281 PMC3632064

[14] Koneczny I Stevens JAA De Rosa A Huda S Huijbers MG Saxena A. IgG4 autoantibodies against muscle-specific kinase undergo fab-arm exchange in myasthenia gravis patients. J Autoimmun. (2017) 77:104–15. doi: 10.1016/j.jaut.2016.11.005, PMID: 27965060

[15] Huijbers MG Vink A-FD Niks EH Westhuis RH van Zwet EW de Meel RH. Longitudinal epitope mapping in MuSK myasthenia gravis: implications for disease severity. J Neuroimmunol. (2016) 291:82–8. doi: 10.1016/j.jneuroim.2015.12.016, PMID: 26857500

[16] Agius MA Twaddle GM Fairclough RH. Epitope spreading in experimental autoimmune myasthenia gravis. Ann N Y Acad Sci. (1998) 841:365–7. doi: 10.1111/j.1749-6632.1998.tb10948.x, PMID: 9668260

[17] Sorrenti B Laurini C Bosco L Strano CMM Scarlato M Gastaldi M. Overcoming therapeutic challenges: successful management of a supposedly triple seronegative, refractory generalized myasthenia gravis patient with efgartigimod. Eur J Neurol. (2024) 31:e16306. doi: 10.1111/ene.16306, PMID: 38716750 PMC11236002

[18] Wiendl H Abicht A Chan A Della Marina A Hagenacker T Hekmat K. Guideline for the management of myasthenic syndromes. Ther Adv Neurol Disord. (2023) 16:17562864231213240. doi: 10.1177/17562864231213240, PMID: 38152089 PMC10752078

[19] Ipe TS Davis AR Raval JS. Therapeutic plasma exchange in myasthenia gravis: a systematic literature review and meta-analysis of comparative evidence. Front Neurol. (2021) 12:662856. doi: 10.3389/fneur.2021.662856, PMID: 34531809 PMC8439193

[20] Pane C Di Stefano V Cuomo N Sarnataro A Vinciguerra C Bevilacqua L. A real-life experience with eculizumab and efgartigimod in generalized myasthenia gravis patients. J Neurol. (2024) 271:6209–19. doi: 10.1007/s00415-024-12588-7, PMID: 39080054 PMC11377599

